# A Pilot Multiplex Salivary Transcriptomic Analysis to Understand the Sex-specific Effects of Maternal Opioid Use in Offspring

**DOI:** 10.21203/rs.3.rs-7418166/v1

**Published:** 2025-09-24

**Authors:** Tomoko Kaneko-Tarui, Francesca Carasi-Schwartz, Kiran Singh, Kelsea R. Gildawie, Fair M. Vassoler, Elizabeth M. Byrnes, Elizabeth Yen

**Affiliations:** Tufts Medical Center; Tufts Medical Center; Tufts Medical Center; Simmons University; Tufts University Cummings School of Veterinary Medicine; Tufts University Cummings School of Veterinary Medicine; Tufts Medical Center

**Keywords:** maternal, offspring, opioid exposure, sex differences, saliva, multiplex transcriptomics

## Abstract

Opioid use disorder affects males and females differently, yet the molecular mechanisms are understudied in neonates. Our laboratory has demonstrated differential sex effects of opioids on the reward and inflammatory pathways related to neonatal feeding behavior that may affect growth and cardiometabolic outcomes. This observational pilot study examined the sex-specific impact of maternal opioid use during pregnancy on reward, energy homeostasis, inflammation, oxidative stress, and neuropathology pathways in offspring. Saliva from nine opioid-exposed and nine non-exposed neonates collected within 48 hours after birth underwent a multiplex, high-throughput analysis of 72 select genes using NanoString’s nCounter^®^ system (NanoString Technologies, Seattle, WA, USA. Despite low RNA abundance in neonatal saliva, experimental conditions were optimized after several trials. Multiplex analysis demonstrated sex-specific molecular effects of maternal opioid use, i.e., upregulated pathways related to reward, inflammation, oxidative stress, feeding, and energy homeostasis pathways in males, and downregulated pathways related to inflammation and cardiovascular function in females. This pilot study demonstrates the feasibility of multiplexing neonatal saliva using a high-throughput platform. Future work will replicate these methods and validate findings in a larger sample to clarify the sex-specific short and long-term impact of maternal opioid use on health outcomes.

## Introduction

The incidence of opioid use disorder (OUD) among pregnant individuals has steadily increased^1,2^, accompanied by a parallel surge in neonatal opioid withdrawal syndrome (NOWS). NOWS affects approximately 6–20 per 1,000 live births, depending on the geographic region and population^3–5^. Medications for opioid use disorder (MOUD), including methadone and buprenorphine, are the standard of care for pregnant individuals with OUD^6,7^. These opioid agonist therapies reduce illicit opioid use, improve maternal health, and promote prenatal care engagement. However, both drugs cross the placenta and can influence fetal neurodevelopment, potentially affecting neonatal feeding behavior, immune function, and long-term health outcomes^8–10^. In addition to the clinical management of acute withdrawal in the period after birth, prenatal opioid exposure is also associated with more chronic and long-term effects, including low birth weight, small-for-gestational-age (SGA) status, impaired postnatal growth, and neurodevelopmental delays^9,11–13^. Therefore, maternal OUD is a significant public health concern, with profound implications for neonatal health and developmental outcomes.

Despite the well-documented clinical manifestations and risks, the molecular mechanisms underlying neonatal responses to opioid exposure *in utero* remain understudied. Emerging evidence from both human and animal studies suggests that sex-specific biological responses may play a critical role in shaping the trajectory of these outcomes. For instance, male neonates exposed to opioids *in utero* are more vulnerable to severe NOWS and exhibit poorer cognitive and behavioral outcomes compared to opioid-exposed females^14,15^. These sex differences are likely driven by variations in immune, metabolic, and neurodevelopmental pathways^16^.

Recent studies have begun to elucidate molecular pathways that may mediate these sex-specific effects of opioid exposure. One such pathway involves the dopamine receptor D2 (DRD2), a critical component of the brain’s reward signaling system^17,18^, which has been implicated in opioid-related reinforcement behaviors. In addition, animal models indicate that opioids can activate toll-like receptor 4 (TLR4) on microglia, triggering proinflammatory cascades that enhance opioid reward and may contribute to neuroinflammation^19,20^. Our laboratory was the first to demonstrate sex-specific effects of maternal opioid use on *DRD2* expression, suggesting a potential mechanism for aberrant feeding behavior in neonates, particularly males^21^. We also demonstrated that upregulation of proinflammatory genes - interleukin-6 (*IL6*), interleukin-1 beta (*IL1β*), and tumor necrosis factor alpha (*TNFα*) - might underlie white matter injury in opioid-exposed offspring, with greater effects observed in females than in males^22^. Together, these findings highlight that reward signaling and immune activation may be differentially regulated by opioids depending on neonatal sex.

Building on these data, the current pilot study aimed to identify additional candidate genes that may address the critical knowledge gap of understanding the sex-specific effects of maternal opioid use. Leveraging multiplex, high-throughput commercial techniques that have not been previously applied to neonatal saliva, we profiled gene pathways related to opioid use, including inflammation, reward, feeding regulation, oxidative stress, and energy metabolism^16–19,21–23^. In addition to opioid exposure and sex, we also explored whether molecular signatures vary by the types of maternal opioid use (methadone, buprenorphine).

## Results

### Participant Characteristics

Following an initial quality check (QC) against the standard for multiplex transcriptomics and further optimization of the transcriptomic experiments (see Methods), samples from 18 neonates (nine opioid-exposed, nine non-exposed) passed the QC and were included in the final analysis. As shown in Table 1, opioid-exposed neonates were more likely to be SGA (33.3% vs. 0%) and born to mothers with hepatitis C (55.6% vs. 0%) compared to non-exposed neonates. Additionally, opioid-exposed neonates were smaller at birth (weight, length, and head circumference). There was a higher proportion of females in the non-exposed group (77.8%) relative to the opioid-exposed group (33.3%).

### Gene Expression Differences by Opioid Exposure

Comparing opioid-exposed with non-exposed neonates, gene expression analyses revealed significant dysregulation in several inflammation- and neurodevelopment-related genes (Table 2). Notably, *PTGS2* (*COX 2*), a key mediator of inflammation, was significantly upregulated in the exposed group (fold change: 3.00, *p* = 0.02). In contrast, expression of *CCL2* and *BDNF* was downregulated (fold changes: − 3.42 and − 2.38, respectively; *p* < 0.05), suggesting suppressed immune regulation and neuronal signaling.

Stratified analyses revealed marked sex-differential effects of maternal opioid use. Within the female cohort, maternal opioid use downregulated inflammation-related genes, including *CCL2*, *CXCR4*, and *IL6* (fold changes: − 8.14 to − 5.29; *p* = 0.01–0.05). In contrast, in the male cohort, maternal opioid use upregulated *DRD2*, *RELA*, *MC4R, FOS, PTGS2*, and *SQSTM1* (fold changes: 3.14 to 6.04; *p* < 0.01–0.05). These genes are implicated in reward signaling, oxidative stress response, feeding and energy regulation, as well as cognition and neurodegenerative disorders. Interestingly, cancer was also involved in several of these pathways.

### Differences by Types of Opioid Exposure

Comparisons between neonates exposed to methadone (n = 2) versus buprenorphine (n = 7) showed that methadone was associated with significantly greater expression of immune-related genes. Specifically, *CXCR4* and *TNF* were upregulated in the methadone group (fold changes: 9.38 and 5.40; *p* < 0.05), as was *SQSTM1*, a gene involved in autophagy, immunity, and neurodegenerative disorders (fold change: 3.14, *p* = 0.02) (Table 2). A few of these pathways were associated with cancer.

### Sex-Specific Differences in Gene Expression

Independent of exposure status, sex-stratified analysis across all neonates showed that males had lower expression of metabolic genes such as *PPARα*, *AKT1*, and *AMPK* compared to females (fold changes: − 4.10 to − 2.46; all *p* < 0.05), and greater expression of prostaglandin biosynthesis factor *PTGS2*, angiogenic and atherosclerotic factor *VEGFα*, and metabolic homeostasis *LEPR* (fold changes: 2.5 to 3.75, *p* < 0.05).

Among non-exposed neonates, *RELA* was downregulated in males compared to females (fold change: − 4.30, *p* = 0.02), suggesting a lower baseline inflammatory state. However, in opioid-exposed neonates, males showed substantial upregulation of inflammatory and immune pathway genes, including *PTGS2*, *TLR4*, *IL1β*, *CXCR4*, and *CXCL8* (fold changes: 2.20 to 6.50; all *p* < 0.05), underscoring a sex-specific activation of immune and neuroinflammatory pathways in response to maternal opioid use. Cancer progression again presented and paralleled the inflammation in some of these pathways (Table 3).

## Discussion

This pilot study provides novel evidence of sex-specific molecular responses in neonates prenatally exposed to maternal methadone and buprenorphine. To our knowledge, this study is the first to leverage a high-throughput, commercial multiomic profiling platform for neonatal saliva using the nCounter^®^ Analysis System (NanoString Technologies, Seattle, WA, USA). By adapting this platform for saliva, we identified distinct patterns in inflammation, reward signaling, and energy regulation pathways, offering insight into the biological mechanisms underlying differential neonatal vulnerability.

Our findings support and extend prior observations from animal models and emerging human data, while introducing neonatal saliva as a feasible biospecimen for molecular analysis. Miller et al. reported reduced neutrophils and inflammatory cytokines in the umbilical cord blood of opioid-exposed neonates compared to those in non-exposed neonates^24^. In contrast, Newville et al. observed elevated proinflammatory cytokines and chemokines, along with heightened immune reactivity, in methadone-exposed rats^25^. Our results integrate these findings by showing that maternal opioid use modulates inflammatory responses in offspring, with the direction and magnitude of changes differing by sex. Specifically, the downregulation of *CCL2* and *BDNF* expression, alongside the upregulation of *PTGS2*, indicates that the inflammatory responses following *in utero* opioid exposure represent a complex interplay of biological and environmental factors. For example, the female-predominant downregulation of CCL2 may underlie the overall reduced expression of this gene in the exposed cohort. In contrast, the increased expression of PTGS2 in exposed males likely drives the elevated levels observed. Thus, sex appears to mediate the inflammatory consequences of maternal opioid use, as evidenced by male-versus female-specific molecular alterations identified in this study. These alterations span pathways related to inflammation, immune regulation, neurodevelopment, cognition, metabolic homeostasis, and neurodegenerative risk.

Therefore, our pilot study highlights the need to include sex as a biological variable in understanding the impact of maternal opioid use on offspring. The greater expression of *DRD2* in opioid-exposed males in the current study aligns with our published data showing the male-predominant effects of opioids on heightened reward signaling and feeding behavior^19^. Of interest is the greater expression of *MC4R* in opioid-exposed males. Given that *MC4R* is critical for feeding and metabolism and is typically upregulated in a satiated state^26^, the concurrent increase in *DRD2* and *MC4R* expression in opioid-exposed neonates suggests that maternal opioid use dysregulates the hypothalamic balance and may explain the feeding dysregulation often observed in these neonates^27^. The elevated expression of *RELA* and *MC4R* further implicates oxidative stress and disrupted energy homeostasis in male neonates, consistent with studies linking prenatal opioid exposure to metabolic dysregulation^27^ and long-term neurobehavioral consequences^9,11–13^. While our data need to be validated in a larger sample size, these findings provide a critical foundation to link sex-specific molecular changes with clinical presentations in opioid-exposed neonates to arrive at effective interventions and personalized care.

Stratification by sex (Table 3) provides further evidence of the distinct molecular response to *in utero* opioid exposure between male and female neonates. Opioid-exposed males exhibited upregulation of inflammation, neuroinflammation, and neurodegenerative disorders as shown by the greater expression of *TLR4, PTGS2, IL1β, CXCR4*, and *CXCL8*. While opioids act via opioid receptors, animal data have demonstrated the non-neuronal effects of opioids by binding with TLR4 in microglia and the release of chemokines and cytokines, and subsequent reinforcement of reward signaling^18^. Our study is the first to suggest that maternal opioid use may modulate inflammation through its sex-specific effect on the TLR4 pathway, which may explain the upregulation of *DRD2* in opioid-exposed males.

In contrast, opioid-exposed females showed marked downregulation of genes in the inflammation and immune pathways. Our findings align with previous evidence that female neonates may possess intrinsic immunological advantages and resilience to prenatal stressors^28^ and that male sex confers a greater vulnerability to adverse prenatal and perinatal effects^29^. The directionality and magnitude of these differences point to a biologically plausible framework in which inflammation and reward circuits are differentially modulated by sex, potentially influencing the severity of NOWS and future neurodevelopment in the offspring.

Another important observation, albeit limited by our small sample size, is the differential gene expression based on the type of maternal opioid use. Neonates exposed to methadone demonstrated upregulation of genes in the inflammatory and immune pathways compared to those exposed to buprenorphine. Based on studies showing worse clinical outcomes in neonates exposed to methadone relative to buprenorphine (e.g., more severe NOWS and more extended hospital stays)^30–32^, the molecular data presented here may suggest a biological basis for these clinical trends.

This study is the first to use neonatal saliva on a high-throughput, commercial transcriptomic platform. Despite the initial assay challenges, our team successfully optimized the experimental conditions to generate the current data. The technical descriptions reported in this study serve as proof of concept that using micro quantities of neonatal saliva to conduct multiplexed gene analyses is feasible and even desirable. Neonatal research is often limited by the types of procedures that can be used to generate data; therefore, the field must leverage the least invasive method possible to ensure safe and robust research in this population.

In addition to the technical novelty, our molecular findings provide intriguing findings that can be utilized in future studies examining the mechanistic and clinical relevance of maternal opioid use in offspring. First, our results reinforce the role of *TLR4* in opioid-related neuroimmune interactions, consistent with animal data showing microglial activation and inflammatory priming following opioid exposure^19,33^. Second, the greater expression of *DRD2* may underscore the higher OUD prevalence in adult males than in females. In other words, the heightened reward signaling early in life may predispose to future reward-seeking behaviors, such as substance use or other types of addiction, with differential sex effects. Third, the consistent enrichment of metabolism-related pathways among opioid-exposed neonates—especially males—needs to be further studied as these early metabolic alterations may contribute to the lower birth weight, aberrant feeding behavior, and growth impairment observed in this population, all of which could lead to cardiometabolic issues in adulthood. Fourth, while the significance of cancer involvement in the pathways affected by maternal opioid use is unclear, future longitudinal studies should examine the risk of malignancy related to *in utero* and childhood opioid exposure. This is especially important given the higher risk of cancer and cancer-related mortality in people with chronic opioid use^34–36^, although a meta-analysis cautioned against the risk of bias in overestimating the overall effect of opioid use on cancer outcomes^36^. Finally, using drops of neonatal saliva, our pilot study highlights the importance of including sex as a biological variable. The current data further support other studies from our group and others, demonstrating the sex-specific risks related to OUD and its impact on offspring. Understanding the dimorphic effects of sex can personalize the care of neonatal opioid withdrawal, which currently is based on a one-size-fits-all paradigm. From a translational perspective, these results highlight the potential for salivary biomarkers to identify neonates at higher risk of adverse outcomes. Such markers could guide personalized care strategies, including closer neurodevelopmental follow-up or early behavioral interventions.

Limitations of the current study include the small sample size, which reduces statistical power and generalizability. The use of saliva, while non-invasive and clinically practical, captures a subset of systemic molecular activity and may not fully reflect brain-specific processes. Additionally, potential confounding by maternal comorbidities (e.g., hepatitis C, polysubstance use) and unmeasured environmental exposures cannot be excluded. Finally, the cross-sectional design precludes conclusions about long-term consequences, underscoring the need for longitudinal follow-up.

Future research should expand sample sizes, include additional tissue types such as cord blood or placenta, neuroimaging data, and developmental screening tests to better understand the associations with neurodevelopmental outcomes, as well as comprehensive cardiometabolic evaluations, including blood pressure and anthropometric measurements. Integrating multi-omics approaches—including epigenetics and proteomics—may further elucidate sex-specific biological programming in opioid-exposed neonates. Importantly, future studies should evaluate whether salivary gene expression profiles can serve as reliable predictors of clinical outcomes or treatment response in neonates with NOWS. Our study also serves as a foundation for a collaboration with preclinical researchers using non-human models to examine the mechanistic underpinnings of maternal opioid use.

In conclusion, maternal opioid use is associated with distinct, sex-specific molecular responses in human neonates, with opioid-exposed males showing increased activation of inflammatory and reward-related pathways, and females displaying relative downregulation of immune signaling. These findings suggest that biological sex plays a critical role in shaping neonatal vulnerability to *in utero* opioid exposure, potentially contributing to the variable severity of NOWS and long-term developmental outcomes.

The observed upregulation of *DRD2* and *TLR4* in opioid-exposed males provides compelling evidence for a mechanistic link between immune activation and altered reward sensitivity, consistent with animal models of opioid reinforcement. Moreover, differential expression patterns based on the type of opioid—methadone versus buprenorphine—highlight the importance of individualized approaches to maternal treatment.

Saliva-based gene expression profiling offers a promising, non-invasive tool to uncover early biomarkers of risk, which may inform sex- and exposure-specific clinical monitoring and intervention strategies. Larger, longitudinal studies are needed to validate these findings and explore their relevance to neurodevelopmental trajectories, ultimately advancing personalized care for opioid-exposed neonates.

## Methods

### Study Design and Participants

This pilot observational study used a prospective cohort design to investigate differences in gene expression between male and female neonates, comparing those with prenatal opioid exposure to those without. A total of 18 neonates were enrolled from the newborn nursery and the neonatal intensive care unit of Tufts Medical Center in Boston, Massachusetts, between February and November 2023. The study cohort included nine neonates with documented maternal opioid use during pregnancy (opioid-exposed group) and nine neonates without such exposure (non-exposed group). Neonates were matched by gestational age to the extent possible.

Inclusion criteria for the opioid-exposed group included: (1) maternal opioid use confirmed by medical record documentation or toxicology screening, and (2) availability of saliva samples collected within 48 hours of birth. The non-exposed group consisted of neonates born to mothers with no known substance use during pregnancy and no detectable opioids in toxicology screening. Exclusion criteria included: major congenital anomalies, perinatal infection, or gestational age < 34 weeks.

Maternal demographic data (including race, ethnicity, delivery type, opioid type, group B streptococcus/GBS colonization status, hepatitis C status, cigarette smoking status) and neonatal clinical characteristics (gestational age, sex, birth weight, length, head circumference and corresponding percentiles, Apgar 1 and 5 minutes, SGA status) were obtained from the medical record.

#### Ethical Approval and Consent to Participate

The study was approved by the Tufts Medical Center Institutional Review Board. All procedures were performed in accordance with institutional and national research committee ethical standards, the Declaration of Helsinki, and relevant guidelines and regulations. Written informed consent for study participation was obtained from a parent or legal guardian of each neonate prior to enrollment. No identifiable images or personal data are included in this manuscript, and all HIPAA identifiers have been removed.

### Sample Collection and RNA Extraction

Saliva samples were collected from neonates within 48 hours of birth using our established techniques^37^. Briefly, saliva was collected using a 1-milliliter (mL) insulin syringe (Becton, Dickinson and Company, Franklin Lakes, NJ) attached to the low-pressure wall suction for 15–30 seconds. To minimize breast milk and associated maternal RNA contamination, saliva was collected before feeding or at least 30 minutes after feeding. Saliva was immediately placed in 250 microliter (μL) RNAprotect Saliva Reagent (Qiagen, Hilden, Germany) to minimize RNA degradation. Total RNA was isolated using the RNeasy Micro Kit (Qiagen, Hilden, Germany) according to the manufacturer’s protocols, which were optimized for low-input, saliva-derived specimens. On-column DNase treatment was performed using RNase-free DNase I (Qiagen) to minimize DNA contamination. Once RNA was extracted, the total RNA was stored at −80°C pending gene expression analysis.

### Gene Expression Analysis

mRNA expression profiling was performed using the NanoString nCounter Analysis System (NanoString Technologies, Seattle, WA), a direct, multiplexed platform for quantifying gene expression. A custom CodeSet was designed to target 72 genes of interest, including selected housekeeping genes used for normalization.

To prepare samples for hybridization, the NanoString Low RNA Input Kit (NanoString Technologies, Seattle, WA) was used according to the manufacturer’s instructions. We subsequently determined that eight pre-amplification cycles were optimal for amplifying the mRNA transcripts using neonatal saliva, which is known to have a low starting mRNA level^38^. The nCounter assay employs a molecular barcoding system, in which specific probes hybridize directly to target mRNAs. Each probe pair includes a capture probe and a reporter probe containing a unique fluorescent barcode for quantification. For each reaction, the pre-amplified RNA was hybridized with the Reporter CodeSet and Capture ProbeSet at 65°C for 16–18 hours, following the standard protocol. Before conducting the main experiments, we performed preliminary optimization tests under varying pre-amplification cycle numbers and hybridization durations. These trials indicated that hybridizing the pre-amplified RNA for 22 hours yielded the most robust and reproducible gene expression profiles.

Data analysis was conducted using the ROSALIND^®^ platform (Rosalind, Inc., San Diego, CA), which employs a HyperScale architecture. As part of quality control, ROSALIND generated read distribution metrics, violin plots, identity heatmaps, and multidimensional scaling (MDS) plots. Normalization, fold change calculation, and statistical testing followed NanoString’s recommended protocols. Specifically, the nCounter Advantage Analysis pipeline was applied, normalizing counts by dividing them by the geometric mean of selected housekeeping genes within each lane. Housekeeping genes confirmed to be expressed in neonatal saliva-GAPDH, HPRT1, and YWHAZ^39^ -were used for normalization.

Differential expression analysis was conducted using a fast methods statistical framework described in the *nCounter Advantage Analysis 2.0 User Manual*, which applies a generalized linear model to estimate fold changes and associated p-values. Multiple testing correction was performed using the Benjamini–Hochberg procedure to control the false discovery rate (FDR). Results with adjusted *p*-values ≥ 0.05 were retained for interpretation. This GLM-based approach is particularly well-suited for NanoString data as it accounts for the discrete nature of count data and accommodates technical variation, thereby enhancing the accuracy and reproducibility of differential expression estimates.

GLMs were prespecified to include the following predictors: sex (male/female), prenatal opioid exposure (unexposed vs exposed), opioid medication type among exposed pregnancies (methadone vs buprenorphine), and whether the infant required postnatal pharmacologic therapy for neonatal opioid withdrawal syndrome (NOWS) (yes/no). For full-cohort analyses, exposure was encoded as a three-level factor (unexposed, methadone, buprenorphine) to avoid collinearity between exposure and medication type. We also conducted an exposed-only sensitivity analysis (methadone vs buprenorphine), adjusting for sex and NOWS therapy. Given the sample size, higher-order interactions were limited to the sex × exposure term, which was specified a priori.

To assess sex-specific gene expression patterns, data were stratified by sex, and differential expression analysis was performed separately for male and female samples. This approach aimed to identify sex-specific differences in gene expression, particularly those potentially influenced by prenatal opioid exposure.

## Figures and Tables

**Table 1:** Demographic Data

	Non-Exposed (N = 9)	Opioid-Exposed (N = 9)	*P*
Female	7 (77.8)	3 (33.3)	0.06
White	7 (77.8)	8 (88.9)	0.59
Non-Hispanic	6 (66.7)	8 (88.9)	0.12
GA (weeks)	37.0 (1.6)	37.6 (1.6)	0.46
BW (grams)	2955.1 (433.8)	2721.4 (624.9)	0.37
BW percentile	62.9 (49.0, 66.0)	28.0 (4.0, 46.0)	**0.02**
Length (cm)	48.3 (2.1)	46.8 (4.5)	0.38
Length percentile	55.0 (37.0, 73.0)	23.0 (10.0, 37.0)	0.07
HC (cm)	33.7 (0.9)	33.0 (2.8)	0.46
HC percentile	74.0 (60.0, 75.0)	21.0 (12.3, 49.0)	0.06
SGA	0 (0.0)	3 (33.3)	**< 0.01**
Cesarean section	6 (66.7)	4 (44.4)	0.34
1-minute Apgar	8.0 (8.0, 9.0)	8.0 (8.0, 8.0)	0.84
5-minute Apgar	9.0 (9.0, 9.0)	9.0 (8.0, 9.0)	0.56
Maternal smoking	1 (11.1)	4 (44.4)	0.09
Maternal Hepatitis C	0 (0.0)	5 (55.6)	**< 0.01**
Maternal GBS	0 (0.0)	2 (22.2)	0.32
Maternal medication	NA	7 (77.8)	NA
Buprenorphine		2 (22.2)	
Methadone			
Polysubstance	NA	6 (66.7)	NA

NA = not available/applicable, GA = gestational age, BW = birth weight, HC = head circumference, SGA = small for gestational age. Data are presented as mean (standard deviation) or median (interquartile ranges) for continuous measures, and N (%) for categorical measures. Bolded p values represent significant differences.

**Table 2:** Differential Gene Expression by Opioid Exposure

By Exposure	Genes	Abbreviation	Pathways/diseases*	Fold Change	P value
All (9 exp. vs 9 non-exp.)	*CCL2*	C-C motif chemokine ligand 2, monocyte chemoattractant protein-1 (*MCP-1*)	Immunoregulatory, inflammation, neurodegenerative disorder, cancer, cardiovascular diseases	−3.42	**0.03**
	*BDNF*	Brain derived neurotrophic factor	Neuronal development, cognition, memory, learning, neuropsychiatry	−2.38	**0.03**
	*PTGS2*	Prostaglandin-endoperoxide synthase 2, cyclooxygenase 2 (*COX 2*)	Inflammation, prostaglandin biosynthesis	3.00	**0.02**
Females (3 exp. vs 7 non-exp.)	*CCL2*	C-C motif chemokine ligand 2, monocyte chemoattractant protein-1 (*MCP-1*)	Immunoregulatory, inflammation, neurodegenerative disorder, cancer, cardiovascular diseases	−8.14	**0.03**
	*CXCR4*	C-X-C motif chemokine receptor 4	Immune response, neurological functions, cancer progression	−6.21	**0.01**
	*IL6*	Interleukin 6	Inflammation, immune response, cancer progression, obesity, diabetes	−5.29	0.05
Males (6 exp. vs 2 non-exp.)	*DRD2*	Dopamine receptor 2	Reward sensitivity, locomotion, cognition, emotion regulation	6.04	0.05
	*RELA*	REL proto-oncogene, NF-κB subunit, p65	Oxidative stress, inflammation, autoimmune disorders, cancer	5.94	**< 0.01**
	*MC4R*	Melanocortin 4 receptor	Energy homeostasis, body weight and feeding regulation, obesity	5.54	0.05
	*FOS*	Fos proto-oncogene	Immune response, inflammation, cancer	4.96	**0.04**
	*PTGS2*	Prostaglandin-endoperoxide synthase 2	Inflammation, prostaglandin biosynthesis	4.33	0.05
	*SQSTM1*	Sequestosome 1 (p62)	Immunity, neurodegenerative disorders, cancer	3.14	**0.02**
By Opioid Type	Genes	Abbreviation	Pathways/diseases*	Fold Change	P value
2 Methadone vs. 7 Buprenorphine	*CXCR4*	C-X-C motif chemokine receptor 4	Immune response, neurological functions, cancer progression	9.38	**< 0.01**
	*TNF*	Tumor necrosis factor	Neuronal development, cognition, memory, learning, neuropsychiatry	5.40	**0.04**
	*SQSTM1*	Sequestosome 1 (p62)	Immunity, neurodegenerative disorders, cancer	3.14	**0.02**

* source: Rosalind^®^ Academy version 3.39.13.1 (https://rosalind.bio/academy); exp: opioid-exposed

**Table 3:** Differential Gene Expression by Sex

By Sex	Genes	Abbreviation	Pathways/diseases*	Fold Change	P value
All (8 males vs 10 females)	*PPARA*	Peroxisome proliferator activated receptor alpha	Energy, cholesterol, and lipid metabolism, glucose homeostasis, immune, inflammation, obesity, atherosclerosis	−4.10	**0.01**
	*AKT1*	AKT serine/threonine kinase 1	Cell metabolism, insulin signaling, cancer,	−3.24	**0.01**
	*AMPK*	AMP-activated protein kinase	Energy metabolism, fatty acid oxidation, inflammation, diabetes, obesity, metabolic disorder	−2.46	**0.04**
	*PTGS2*	Prostaglandin-endoperoxide synthase 2	Inflammation, prostaglandin biosynthesis	3.75	**< 0.01**
	*VEGFA*	Vascular endothelial growth factor A	Angiogenesis, atherosclerosis, hypoxic-induced neovascularization, obesity, tumor growth, cancer	3.15	**0.03**
	*LEPR*	Leptin receptor	Energy homeostasis, metabolism, insulin signaling, glucose homeostasis, obesity, cancer, metabolic syndrome, cardiovascular disease	2.5	**0.03**
Non-exp. (2 males vs 7 females)	*RELA*	REL proto-oncogene, NFκB subunit, p65	Oxidative stress, inflammation, autoimmune disorders, cancer	−4.30	**0.02**
Exp. (6 males vs 3 females)	*PTGS2*	Prostaglandin-endoperoxide synthase 2, cyclooxygenase 2 (*COX 2*)	Inflammation, prostaglandin biosynthesis	6.50	**0.01**
	*TLR4*	Toll-like receptor 4	Innate immunity, pathogen recognition, inflammation, microglia activation, neuroinflammation, cancer, autoimmune disorders, neurodegeneration	5.88	0.05
	*IL1B*	Interleukin 1 beta	Inflammation, neuroinflammation, cancer, diabetes, bipolar disorder, Alzheimer’s disease	5.49	**0.04**
	*CXCR4*	C-X-C motif chemokine receptor 4	Immune response, neurological functions, cancer progression	2.29	**0.03**
	*CXCL8*	C-X-C motif ligand 8	Inflammation, cancer, neurodegeneration, neuroinflammation, Parkinson’s disease	2.20	0.05

* source: Rosalind^®^ Academy version 3.39.13.1 (https://rosalind.bio/academy); exp: opioid-exposed

## Data Availability

The datasets generated during and/or analyzed during the current study are available from the corresponding author on reasonable request.

## References

[1] Jarlenski M. Healthcare Patterns of Pregnant Women and Children Affected by OUD in 9 State Medicaid Populations. J. Addict. Med. 15, 406–413 (2021). 33560699 10.1097/ADM.0000000000000780 PMC8339176

[2] Klaman S. L. Treating Women Who Are Pregnant and Parenting for Opioid Use Disorder and the Concurrent Care of Their Infants and Children. J. Addict. Med. 11, 178–190 (2017). 28406856 10.1097/ADM.0000000000000308 PMC5457836

[3] Patrick S. W. Neonatal Abstinence Syndrome and Associated Health Care Expenditures: United States, 2000–2009. JAMA 307, 1934–1940 (2012). 22546608 10.1001/jama.2012.3951

[4] Huybrechts K. F. Risk of neonatal drug withdrawal after intrauterine co-exposure to opioids and psychotropic medications: cohort study. BMJ 358, j3326 (2017). 28768628 10.1136/bmj.j3326 PMC5538591

[5] Leech A. A., Cooper W. O., McNeer E., Scott T. A., & Patrick S. W. Neonatal Abstinence Syndrome In The United States, 2004–16. Heal. Aff. 39, 764–767 (2020). 10.1377/hlthaff.2019.00814 PMC7733618 32364857

[6] Miele K. Medication for Opioid Use Disorder During Pregnancy — Maternal and Infant Network to Understand Outcomes Associated with Use of Medication for Opioid Use Disorder During Pregnancy (MAT-LINK), 2014–2021. MMWR Surveill. Summ. 72, 1–14 (2023). 10.15585/mmwr.ss7203a1 PMC10154076 37130060

[7] Committee Opinion No. 711. Obstet. Gynecol. 130, e81–e94 (2017). 28742676 10.1097/AOG.0000000000002235

[8] Andersen J. M., Høiseth G. & Nygaard E. Prenatal exposure to methadone or buprenorphine and long-term outcomes: A meta-analysis. Early Hum. Dev. 143, 104997 (2020). 32146140 10.1016/j.earlhumdev.2020.104997

[9] Mactier H. & Hamilton R. Prenatal opioid exposure – Increasing evidence of harm. Early Hum. Dev. 150, 105188 (2020). 32958331 10.1016/j.earlhumdev.2020.105188

[10] Brogly S. B., Saia K. A., Walley A. Y., Du H. M. & Sebastiani P. Prenatal Buprenorphine Versus Methadone Exposure and Neonatal Outcomes: Systematic Review and Meta-Analysis. Am. J. Epidemiology 180, 673–686 (2014). 10.1093/aje/kwu190 25150272

[11] Azuine R. E. Prenatal Risk Factors and Perinatal and Postnatal Outcomes Associated With Maternal Opioid Exposure in an Urban, Low-Income, Multiethnic US Population. JAMA Netw. Open 2, e196405 (2019). 31251378 10.1001/jamanetworkopen.2019.6405 PMC6604084

[12] VELEZ M. L., JANSSON L. M., SCHROEDER J. & WILLIAMS E. Prenatal Methadone Exposure and Neonatal Neurobehavioral Functioning. Pediatr. Res. 66, 704–709 (2009). 19690513 10.1203/PDR.0b013e3181bc035d PMC2796281

[13] Velez M. L. Prenatal buprenorphine exposure and neonatal neurobehavioral functioning. Early Hum. Dev. 117, 7–14 (2018). 29223912 10.1016/j.earlhumdev.2017.11.009

[14] Charles M. K. Male Sex Associated With Increased Risk of Neonatal Abstinence Syndrome. Hosp. Pediatr. 7, 328–334 (2017). 28465360 10.1542/hpeds.2016-0218 PMC5519405

[15] O’Connor A. B., O’Brien L. & Alto W. A. Are there gender related differences in neonatal abstinence syndrome following exposure to buprenorphine during pregnancy? jpme 41, 621–623 (2013). 10.1515/jpm-2012-0288 23612625

[16] McCarthy M. M. Sex differences in the developing brain as a source of inherent risk. Dialogues Clin. Neurosci. 18, 361–372 (2016). 28179808 10.31887/DCNS.2016.18.4/mmccarthy PMC5286722

[17] Maldonado R. Absence of opiate rewarding effects in mice lacking dopamine D2 receptors. Nature 388, 586–589 (1997). 9252189 10.1038/41567

[18] Noble E. P. Addiction and its reward process through polymorphisms of the D2 dopamine receptor gene: a review. Eur. Psychiatry 15, 79–89 (2000). 10.1016/s0924-9338(00)00208-x 10881203

[19] Zhang P. Toll-Like Receptor 4 (TLR4)/Opioid Receptor Pathway Crosstalk and Impact on Opioid Analgesia, Immune Function, and Gastrointestinal Motility. Front. Immunol. 11, 1455 (2020). 32733481 10.3389/fimmu.2020.01455 PMC7360813

[20] King’uyu D. N. The effect of morphine on rat microglial phagocytic activity: An in vitro study of brain region-, plating density-, sex-, morphine concentration-, and receptor-dependency. J. Neuroimmunol. 384, 578204 (2023). 37774553 10.1016/j.jneuroim.2023.578204

[21] Yen E. Sex-Dependent Gene Expression in Infants with Neonatal Opioid Withdrawal Syndrome. J Pediatrics 214, 60–65.e2 (2019). 10.1016/j.jpeds.2019.07.032 PMC10564583 31474426

[22] Yen E. Sex-specific inflammatory and white matter effects of prenatal opioid exposure: a pilot study. Pediatr Res 1–8 (2022) doi:10.1038/s41390-022-02357-5. 36280708 PMC9998341

[23] Elman I. Metabolic and Addiction Indices in Patients on Opioid Agonist Medication-Assisted Treatment: A Comparison of Buprenorphine and Methadone. Sci. Rep. 10, 5617 (2020). 32221389 10.1038/s41598-020-62556-0 PMC7101411

[24] Miller N. W. The impact of opioid exposure during pregnancy on the human neonatal immune profile. Pediatr. Res. 92, 1566–1574 (2022). 35288639 10.1038/s41390-022-02014-x PMC8920062

[25] Newville J., Maxwell J. R., Kitase Y., Robinson S. & Jantzie L. L. Perinatal Opioid Exposure Primes the Peripheral Immune System Toward Hyperreactivity. Front. Pediatr. 8, 272 (2020). 32670993 10.3389/fped.2020.00272 PMC7332770

[26] Ross R. A. Prefrontal cortex melanocortin 4 receptors (MC4R) mediate food intake behavior in male mice. Physiol. Behav. 269, 114280 (2023). 37369302 10.1016/j.physbeh.2023.114280 PMC10528493

[27] Yen E. & Maron J. L. Aberrant Feeding and Growth in Neonates With Prenatal Opioid Exposure: Evidence of Neuromodulation and Behavioral Changes. Front. Pediatr. 9, 805763 (2022). 35127598 10.3389/fped.2021.805763 PMC8814597

[28] Nugent B. M., O’Donnell C. M., Epperson C. N. & Bale T. L. Placental H3K27me3 establishes female resilience to prenatal insults. Nat. Commun. 9, 2555 (2018). 29967448 10.1038/s41467-018-04992-1 PMC6028627

[29] DiPietro J. A. & Voegtline K. M. The gestational foundation of sex differences in development and vulnerability. Neuroscience 342, 4–20 (2017). 26232714 10.1016/j.neuroscience.2015.07.068 PMC4732938

[30] E. J. H. Neonatal Abstinence Syndrome after Methadone or Buprenorphine Exposure. N. Engl. J. Med. 363, 2320–2331 (2010). 21142534 10.1056/NEJMoa1005359 PMC3073631

[31] Hall E. S. A Cohort Comparison of Buprenorphine versus Methadone Treatment for Neonatal Abstinence Syndrome. J. Pediatr. 170, 39–44.e1 (2016). 26703873 10.1016/j.jpeds.2015.11.039

[32] Jones H. E. Buprenorphine versus methadone in the treatment of pregnant opioid-dependent patients: effects on the neonatal abstinence syndrome. Drug Alcohol Depend.79, 1–10 (2005). 15943939 10.1016/j.drugalcdep.2004.11.013

[33] Green J. M., Sundman M. H. & Chou Y. Opioid-induced microglia reactivity modulates opioid reward, analgesia, and behavior. Neurosci. Biobehav. Rev. 135, 104544 (2022). 35090951 10.1016/j.neubiorev.2022.104544 PMC9699693

[34] Sheikh M. Opium use and subsequent incidence of cancer: results from the Golestan Cohort Study. Lancet Glob. Heal. 8, e649–e660 (2020). 10.1016/S2214-109X(20)30059-0 PMC7196888 32353313

[35] Sun M., Lin J.-A., Chang C.-L., Wu S.-Y. & Zhang J. Association between long-term opioid use and cancer risk in patients with chronic pain: a propensity score-matched cohort study. Br. J. Anaesth. 129, 84–91 (2022). 35597621 10.1016/j.bja.2022.04.014

[36] Shrestha S., Foot H., Sheikh M., Parat M.-O. & Caze A. L. Opioid use and the risk of cancer incidence and mortality: a systematic review. Cancer Metastasis Rev. 44, 54 (2025). 40498363 10.1007/s10555-025-10268-0 PMC12159095

[37] Dietz J. A., Johnson K. L., Wick H. C., Bianchi D. W. & Maron J. L. Optimal Techniques for mRNA Extraction from Neonatal Salivary Supernatant. Neonatology 101, 55 60 (2012). 21791940 10.1159/000328026 PMC3151004

[38] Yen E., Kaneko-Tarui T. & Maron J. L. Technical Considerations and Protocol Optimization for Neonatal Salivary Biomarker Discovery and Analysis. Frontiers Pediatrics 8, 618553 (2021). 10.3389/fped.2020.618553 PMC7870796 33575231

[39] Khanna P., Johnson K. L. & Maron J. L. Optimal reference genes for RT-qPCR normalization in the newborn. Biotech Histochem 92, 459 466 (2017). 28910197 10.1080/10520295.2017.1362474

